# Prognostic values of the clinicopathological characteristics and survival outcomes in micropapillary urothelial carcinoma of the bladder: A SEER database analysis

**DOI:** 10.1002/cam4.3147

**Published:** 2020-06-11

**Authors:** Di Jin, Kun Jin, Shi Qiu, Xianghong Zhou, Qiming Yuan, Lu Yang, Qiang Wei

**Affiliations:** ^1^ Department of Urology Institute of Urology National Clinical Research Center for Geriatrics and Center of Biomedical Big Data West China Hospital of Sichuan University Chengdu Sichuan Province China

**Keywords:** micropapillary urothelial carcinoma, prognosis, SEER Program, urinary bladder

## Abstract

**Purpose:**

To study prognostic values of the clinicopathological characteristics and survival outcomes in micropapillary urothelial carcinoma (MPUC) of the urinary bladder.

**Method:**

We used the national Surveillance, Epidemiology, and End Results database (2004‐2016) to compare MPUC with transitional cell carcinoma (TCC) and to investigate prognostic values of clinicopathological characteristics, as well as survival outcomes, in MPUC of the urinary bladder. A multivariable Cox proportional hazard model, subgroup analyses, and propensity score matching were used.

**Results:**

In all, 519 patients with MPUC and 154 453 patients with TCC were enrolled. Compared with TCC, patients with MPUC had a higher rate of muscle invasive disease (*P* < .001), lymph node metastasis (*P* < .001), and distal metastasis (*P* < .001), as well as higher tumor grade (*P* < .001). According to the survival analyses, the MPUC group also had lower survival probability in both cancer‐specific mortality (CSM) (*P* < .0001) and overall mortality (OM) analyses (*P* < .0001). Cox proportional hazard regression showed that the MPUC group had a higher risk of OM (hazard ratios [HR] = 1.39, 95% confidence intervals [CI] = 1.22‐1.57, *P* < .0001), although the CSM (HR = 1.18, 95% CI = 1.00‐1.40, *P* = .0505) in that group was fair. In the subgroup analysis, only MPUC patients without distal metastasis faced a higher risk of CSM (HR = 1.33, 95% CI = 1.101.61, *P* < .0001).

**Conclusions:**

Micropapillary urothelial carcinoma prognosis is poorer than that of TCC. Micropapillary urothelial carcinoma is an independent prognostic factor for OM in patients with urinary bladder cancer.

## Graphical abstract

The prognosis of micropapillary urothelial carcinoma (MPUC) is poorer than transitional cell carcinoma. Micropapillary urothelial carcinoma is an independent prognostic factor for overall mortality to patients with urinary bladder cancer. In subgroup analysis, only none distal metastasis MPUC patients faced higher risk of cancer‐specific mortality.

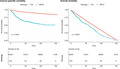

## 1 INTRODUCTION

Bladder cancer is one of the most common cancer types and is a significant cause of tumor‐related death worldwide.^1^ The worldwide age‐standardized incidence rate (per 100 000 person/y) of bladder cancer is 9.0 for men and 2.2 for women.^2^ The most common pathological type is transitional cell carcinoma (TCC),^3^ and thus, treatments for bladder cancer focus primarily on TCC. Since Amin et al reported a micropapillary component in TCC of the urinary bladder and its poor prognosis in 1994.^4^ The incidence of micropapillary urothelial carcinoma (MPUC) is 0.7%‐8.3%, according to various published articles.^5^, ^6^ Micropapillary urothelial carcinoma is characterized by small, tight clusters of high‐grade tumor cells that lack true fibrovascular cores and are contained within lacunar spaces; thus, it often has an aggressive clinical course.^7^ However, MPUC histology as an independent prognostic factor is still controversial. According to Sui et al's analysis of the National Cancer Database, MPUC has a poor prognosis regardless of treatment modality.^8^ However, the multi‐institutional analysis by Mitra et al found that even MPUC is associated with advanced disease at cystectomy, but the clinical outcomes are similar to those of pure TCC after controlling for pathologic features.^9^ Moreover, a meta‐analysis supports the finding that patients with MPUC who undergo radical cystectomy (RC) have survival outcomes similar to those of patients with TCC.^10^ Thus far, given MPUC's rarity, there is no analysis based on sufficient sample size. Therefore, we used the national Surveillance, Epidemiology, and End Results (SEER) database (2004‐2016) to investigate prognostic values of clinicopathological characteristics and survival outcomes in MPUC of the urinary bladder.

## 2 MATERIALS AND METHODS

### 2.1 Data resource and study population

Adults patients (≥18 years of age) who were registered from 2004 to 2016 in the SEER database were selected. The primary cancer site was restricted to the urinary bladder according to the International Classification of Disease for Oncology, Third Edition. Patients were included only if the histology was MPUC or TCC. The diagnosis was confirmed by positive histology and was their first or only cancer diagnosis (first positive indicator of malignancy).

### 2.2 End points

The main end points were overall mortality (OM) and cancer‐specific mortality (CSM) according to data in the SEER database. Overall mortality refers to deaths from any cause, while CSM is defined as death from MPUC or TCC according to the recorded cause of death. Survival time was the duration from initial diagnosis to death from any cause or to the last follow‐up.

### 2.3 Statistical analysis

Baseline characteristics were assessed to determine whether there were significant differences in the distribution of the study population. Two‐sample *t* tests and Pearson's chi‐square tests were performed for continuous variables and categorical variables, respectively. Continuous variables were presented as the mean ± SD. For age at diagnosis and survival (in months), medians and interquartile ranges were also reported. Categorical variables were shown as frequencies and their proportions. The OM and CSM of each histological subtype were compared using unadjusted Kaplan‐Meier curves and the log‐rank test.

The multivariable Cox proportional hazard model was used to calculate hazard ratios (HR) and their 95% confidence intervals (95% CI) stratified by histological types. The following covariates were adjusted: sex, age at diagnosis, primary site, treatment modality (surgery and radiation), and tumor‐node‐metastasis (TNM) stage. Subgroup analyses were performed by multivariate regression analysis. Sex, age at diagnosis, TNM stage, and treatment modality (surgery and radiation) were adjusted in the Cox model. Tests to determine interactions were also used in the subgroup analyses. Propensity score matching (PSM) was used to further adjust the model for potential baseline confounding factors. All analyses were performed with the statistical software packages R (http://www.R‐project.org; The R Foundation) and EmpowerStats (http://www.empowerstats.com; X&Y Solutions, Inc).

## 3 RESULTS

### 3.1 Baseline characteristics of the study population

In all, 154 972 patients were diagnosed with TCC and MPUC in the SEER database from 2004 to 2016. This study included 154 453 patients with TCC and 519 patients with MPUC were included. Table 1 includes the patients' baseline characteristics. At diagnosis, patients with MPUC were close in age to those with TCC (MPUC 71.16 ± 10.91 vs TCC 70.91 ± 12.06, *P* = .628). Most patients were males in both the MPUC (80.54%) and TCC (76.97%) groups, and there was no difference in the proportion of males and females between the two groups (*P* = .315). Patients in the MPUC group presented at a more advanced stage than those in the TCC group, as shown by a higher rate of muscle invasive disease (63.39% vs 9.39%, *P* < .001), lymph node metastasis (24.28% vs 1.32%, *P* < .001), and distal metastasis (10.79% vs 1.17%, *P* < .001). Higher‐grade disease was also more common in the MPUC group (97.49% vs 49.38%, *P* < .001). The surgery constituent ratio was significantly different between the two groups (*P* < .001), and patients in the MPUC group were more likely to undergo RC (19.27% vs 2.68%) and pelvic exenteration (17.34% vs 1.56%). Moreover, lymph nodes were more likely to be removed from patients in the MPUC group than from patients in the TCC group (38.54% vs 4.24%, *P* < .001). Moreover, regarding known radiation therapy, beam radiation was used more frequently in the MPUC group than in the TCC group (11.18% vs 2.00%, *P* < .001).

**TABLE 1:** Baseline demographic and clinicopathologic characteristics of patients with MPUC compared to TCC

	MPUC (n = 519)	TCC (n = 154 453)	*P*‐value
Mean age (y, SD)	71.16 ± 10.91	70.91 ± 12.06	.628
Median age (y, IQR)	71.00 (64.00‐79.00)	72.00 (63.00‐80.00)	.866
Sex			.054
Male	418 (80.54%)	118 876 (76.97%)	
Female	101 (19.46%)	35 577 (23.03%)	
Marital status			.065
Married	322 (62.04%)	92 245 (59.72%)	
Single	60 (11.56%)	15 571 (10.08%)	
Widowed/Divorced	108 (20.81%)	33 334 (21.58%)	
Unknown	29 (5.59%)	13 303 (8.61%)	
Race			.164
White	462 (89.02%)	138 377 (89.59%)	
Black	30 (5.78%)	7584 (4.91%)	
Other	25 (4.82%)	6368 (4.12%)	
Unknown	2 (0.39%)	2124 (1.38%)	
Year of diagnosis			<.001
2004	12 (2.31%)	11 581 (7.50%)	
2005	11 (2.12%)	11 076 (7.17%)	
2006	23 (4.43%)	11 365 (7.36%)	
2007	23 (4.43%)	11 745 (7.60%)	
2008	26 (5.01%)	11 643 (7.54%)	
2009	44 (8.48%)	11 632 (7.53%)	
2010	42 (8.09%)	11 919 (7.72%)	
2011	36 (6.94%)	11 794 (7.64%)	
2012	42 (8.09%)	12 299 (7.96%)	
2013	48 (9.25%)	12 157 (7.87%)	
2014	68 (13.10%)	12 341 (7.99%)	
2015	67 (12.91%)	12 534 (8.12%)	
2016	77 (14.84%)	12 367 (8.01%)	
Primary site			<.001
Trigone of bladder	27 (5.20%)	9944 (6.44%)	
Dome of bladder	27 (5.20%)	4541 (2.94%)	
Lateral wall of bladder	97 (18.69%)	34 216 (22.15%)	
Anterior wall of bladder	9 (1.73%)	2861 (1.85%)	
Posterior wall of bladder	44 (8.48%)	14 551 (9.42%)	
Bladder neck	11 (2.12%)	4481 (2.90%)	
Ureteric orifice	5 (0.96%)	6986 (4.52%)	
Urachus	1 (0.19%)	25 (0.02%)	
Overlapping lesion of bladder	85 (16.38%)	14 142 (9.16%)	
Bladder, NOS	213 (41.04%)	62 706 (40.60%)	
T stage			<.001
Ta	18 (3.47%)	99 586 (64.48%)	
Tis	5 (0.96%)	2450 (1.59%)	
T1	164 (31.60%)	36 159 (23.41%)	
T2	195 (37.57%)	10 394 (6.73%)	
T3	75 (14.45%)	2014 (1.30%)	
T4	59 (11.37%)	2095 (1.36%)	
Unknown	3 (0.58%)	1755 (1.14%)	
N stage			<.001
N0	384 (73.99%)	149 639 (96.88%)	
N1	32 (6.17%)	992 (0.64%)	
N2	86 (16.57%)	979 (0.63%)	
N3	8 (1.54%)	72 (0.05%)	
Unknown	9 (1.73%)	2771 (1.79%)	
M stage			<.001
M0	459 (88.44%)	150 922 (97.71%)	
M1	56 (10.79%)	1810 (1.17%)	
Unknown	4 (0.77%)	1721 (1.11%)	
Grade			<.001
Low	9 (2.51%)	39 365 (50.62%)	
High	349 (97.49%)	38 407 (49.38%)	
Surgery			<.001
No surgery	17 (3.28%)	8192 (5.30%)	
TURBT	244 (47.01%)	111 962 (72.49%)	
Partial cystectomy	9 (1.73%)	1134 (0.73%)	
Radical cystectomy	100 (19.27%)	4143 (2.68%)	
Pelvic exenteration	90 (17.34%)	2404 (1.56%)	
Other	58 (11.18%)	26 357 (17.06%)	
Unknown procedure	1 (0.19%)	261 (0.17%)	
Lymph nodes removed			<.001
None	319 (61.46%)	147 899 (95.76%)	
More than one	200 (38.54%)	6554 (4.24%)	
Radiation			<.001
Beam radiation	58 (11.18%)	3094 (2.00%)	
Radioactive implants	0 (0.00%)	12 (0.01%)	
Combination of beam and implants	0 (0.00%)	2 (0.00%)	
Radioisotopes	0 (0.00%)	11 (0.01%)	
Radiation unknown	0 (0.00%)	68 (0.04%)	
Performance unknown	461 (88.82%)	151 266 (97.94%)	
Cancer‐specific mortality			<.001
Alive	373 (71.87%)	141 802 (91.81%)	
Dead	146 (28.13%)	12 651 (8.19%)	
Overall mortality			<.001
Alive	268 (51.64%)	54 682 (35.40%)	
Dead	251 (48.36%)	99 771 (64.60%)	
Survival time (y, SD)	31.76 (33.08)	54.94 (42.02)	<.001
Survival time (y, IQR)	19.00 (8.00‐44.00)	46.00 (19.00‐85.00)	<.001

Abbreviations: IQR, interquartile range; MPUC, micropapillary urothelial carcinoma; NOS, not otherwise specified; TCC, transitional cell carcinoma; TURBT, transurethral resection of bladder tumor.

### 3.2 Survival analyses

**FIGURE 1:**
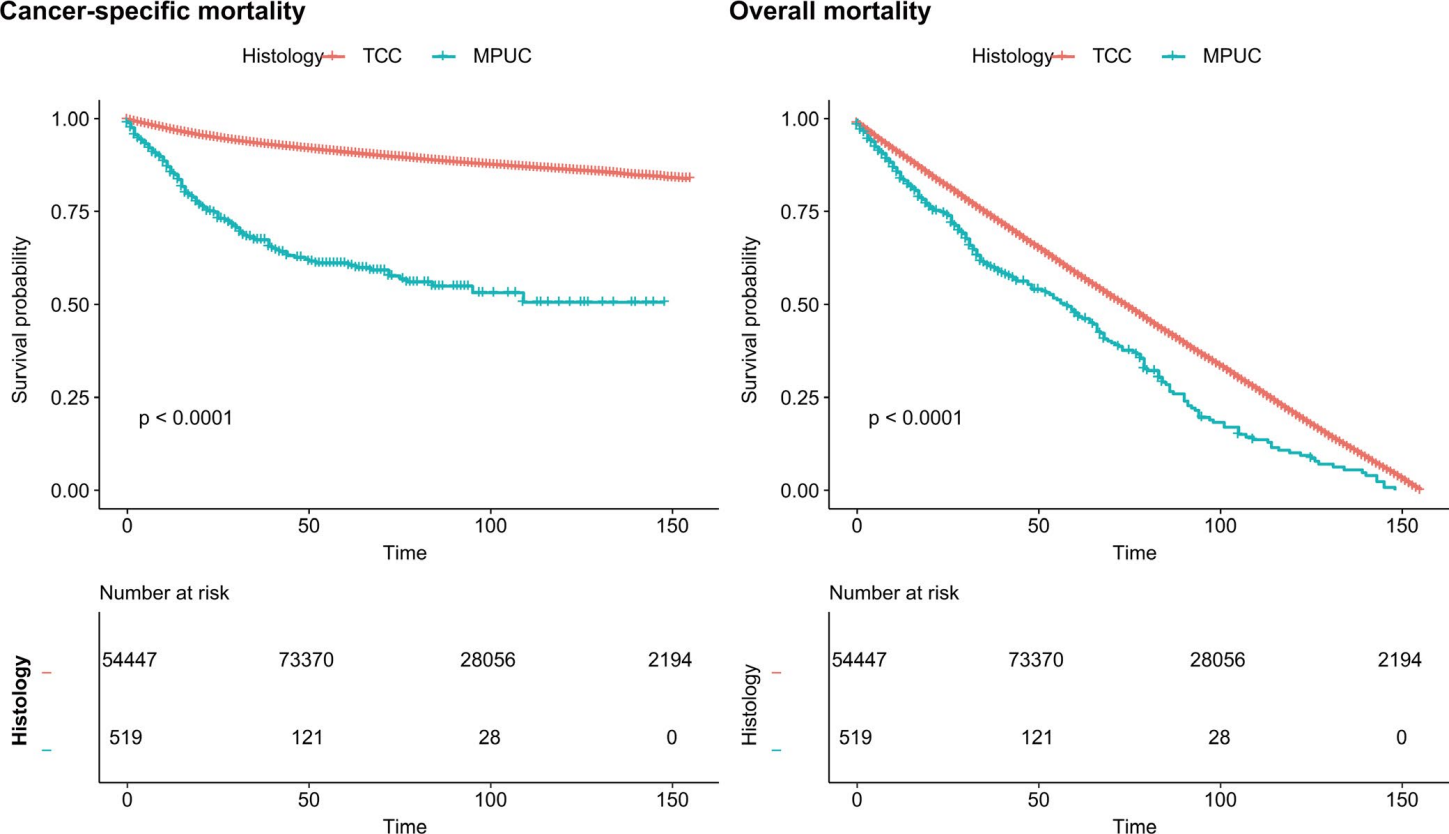
Cancer‐specific mortality and overall mortality of patients with micropapillary urothelial carcinoma (MPUC) and transitional cell carcinoma (TCC) respectively

In survival analyses, the overall survival probability of patients in the MPUC group declined significantly faster than that of patients in the TCC group (*P* < .0001; Figure 1). When the landmark was set at 5 years (60 months), the survival probability of the MPUC group also declined faster in the OM analyses (Figure S1). The MPUC group also had a lower survival probability in the CSM analyses (*P* < .0001; Figure 1).

Table 2 presents multivariable Cox proportional hazard models. After adjustments for age, sex, TNM stage, tumor site, and treatment method, the adjusted model II showed that the MPUC group had a significantly higher risk of OM compared with the TCC group (HR = 1.39, 95% CI = 1.22‐1.57, *P* < .0001), while no difference in CSM was observed between the two groups (HR = 1.18, 95% CI = 1.00‐1.40, *P* = .0505). To minimize selection bias, PSM was performed for baseline factors and treatments (Table 3). However, there were still differences in N stage, M stage, grade, surgery, and lymph nodes removed between the groups. Furthermore, we performed an extra adjustment to analyze the mismatched baseline factors. In the PSM adjusted model I, the MPUC group did not show a higher risk of OM (HR = 1.09, 95% CI = 0.92‐0.29, *P* = .3097) or CSM (HR = 1.18, 95% CI = 0.92‐1.51, *P* = .2049). After further adjustment for T stage and radiation, the PSM adjusted model II showed that the MPUC group faced higher risks of CSM (HR = 1.30, 95% CI = 1.00‐1.67, *P* = .0469), but there was no difference in OM (HR = 1.10, 95% CI = 0.93‐1.31, *P* = .2688).

**TABLE 2:** Multivariable Cox proportional hazard model

Outcomes	MPUC HR (95% CI)	*P*‐value
Overall mortality		
Non‐adjusted	1.50 (1.33, 1.70)	<.0001
Adjusted I	1.40 (1.24, 1.59)	<.0001
Adjusted II	1.39 (1.22, 1.57)	<.0001
PSM non‐adjusted	1.48 (1.29, 1.71)	<.0001
PSM adjusted	1.10 (0.93, 1.31)	.2688
Cancer‐specific mortality		
Non‐adjusted	5.20 (4.42, 6.12)	<.0001
Adjusted I	1.06 (0.90, 1.25)	.5044
Adjusted II	1.18 (1.00, 1.40)	.0505
PSM non‐adjusted	1.35 (1.12, 1.62)	.0016
PSM adjusted	1.30 (1.00, 1.67)	.0469

Note

Adjusted I model adjust for: T stage; N stage; M stage.

Adjusted II model adjust for: Sex; Age; Primary Site; T stage; N stage; M stage; surgery; radiation.

PSM non‐adjusted model adjust for none.

PSM adjusted model adjust for: T stage; N stage; M stage; surgery; radiation; grade; lymph nodes removed.

Abbreviations: HR, hazard ratio; MPUC, micropapillary urothelial carcinoma; PSM, propensity score matching.

**TABLE 3:** Propensity score matching for baseline factors

	MPUC (n = 519)	TCC (n = 1996)	Standardized difference	*P*‐value
Mean Age (y, SD)	71.06 ± 10.90	71.01 ± 11.40	0.0046	.9280
Sex			0	1.0000
Male	401 (80.4)	1604 (80.4)		
Female	98 (19.6)	392 (19.6)		
Marital status				.2326
Married	312 (62.5)	1149 (57.6)	0.1014	
Single	58 (11.6)	247 (12.4)	0.0231	
Widowed/Divorced	101 (20.2)	470 (23.5)	0.0800	
Unknown	28 (5.6)	130 (6.5)	0.0378	
Race				.8007
White	442 (88.6)	1776 (89)	0.0127	
Black	30 (6)	125 (6.3)	0.0104	
Other	25 (5)	83 (4.2)	0.0407	
Unknown	2 (0.4)	12 (0.6)	0.0284	
Primary site				.1086
Trigone of bladder	26 (5.2)	89 (4.5)	0.0350	
Dome of bladder	26 (5.2)	73 (3.7)	0.0755	
Lateral wall of bladder	96 (19.2)	347 (17.4)	0.0479	
Anterior wall of bladder	9 (1.8)	55 (2.8)	0.0638	
Posterior wall of bladder	42 (8.4)	153 (7.7)	0.0276	
Bladder neck	11 (2.2)	63 (3.2)	0.059	
Ureteric orifice	5 (1)	51 (2.6)	0.1177	
Urachus	1 (0.2)	1 (0.1)	0.0425	
Overlapping lesion of bladder	81 (16.2)	283 (14.2)	0.0572	
Bladder, NOS	202 (40.5)	881 (44.1)	0.0741	
T stage				.0931
Ta	18 (3.6)	72 (3.6)	0	
Tis	4 (0.8)	16 (0.8)	0	
T1	156 (31.3)	648 (32.5)	0.0258	
T2	193 (38.7)	771 (38.6)	0.0010	
T3	71 (14.2)	244 (12.2)	0.0592	
T4	55 (11)	194 (9.7)	0.0427	
Unknown	2 (0.4)	51 (2.6)	0.1792	
N stage				<.0001
N0	372 (74.5)	1535 (76.9)	0.0549	
N1	29 (5.8)	116 (5.8)	0	
N2	81 (16.2)	215 (10.8)	0.1603	
N3	8 (1.6)	13 (0.7)	0.0903	
Unknown	9 (1.8)	117 (5.9)	0.2126	
M stage				.0269
M0	444 (89)	1788 (89.6)	0.0194	
M1	51 (10.2)	160 (8)	0.0766	
Unknown	4 (0.8)	48 (2.4)	0.1279	
Grade			0.3848	<.0001
Low	6 (1.7)	112 (11)		
High	337 (98.3)	909 (89)		
Surgery				.0001
No surgery	16 (3.2)	92 (4.6)	0.0724	
TURBT	233 (46.7)	1115 (55.9)	0.1842	
Partial cystectomy	9 (1.8)	30 (1.5)	0.0236	
Radical cystectomy	97 (19.4)	336 (16.8)	0.0677	
Pelvic exenteration	86 (17.2)	201 (10.1)	0.2098	
Other	57 (11.4)	218 (10.9)	0.0159	
Unknown procedure	1 (0.2)	4 (0.2)	0	
Lymph nodes removed			0.2643	<.0001
None	306 (61.3)	1469 (73.6)		
More than one	193 (38.7)	527 (26.4)		
Radiation				.1759
Beam radiation	58 (11.6)	183 (9.2)	0.0805	
Radiation unknown	0 (0)	3 (0.2)	0.0549	
Performance unknown	441 (88.4)	1810 (90.7)	0.0753	
Cancer‐specific mortality				.0625
Alive	353 (70.7)	1496 (74.9)	0.0947	
Dead	146 (29.3)	500 (25.1)		
Overall mortality			0.0411	.4400
Alive	256 (51.3)	1065 (53.4)		
Dead	243 (48.7)	931 (46.6)		

Abbreviations: MPUC, micropapillary urothelial carcinoma; NOS, not otherwise specified; TCC, transitional cell carcinoma; TURBT, transurethral resection of bladder tumor.

### 3.3 Subgroup analyses

**FIGURE 2:**
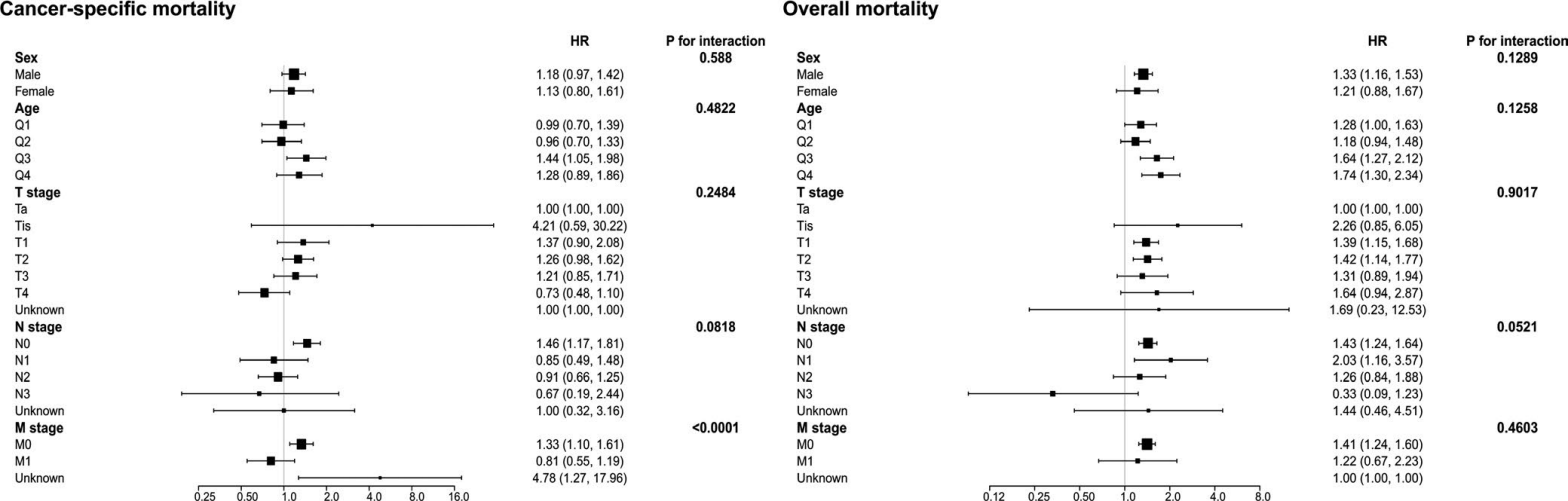
Subgroup analysis for interaction between micropapillary urothelial carcinoma and potential covariates in both overall mortality and cancer‐specific mortality

The subgroup analytical results are shown in Figure 2. After adjusting for potential covariates, the tests for interaction were not statistically significant for sex, age, T stage, and N stage in terms of both OM and CSM. This indicated that MPUC had a worse prognosis in all groups except for distal metastasis. Only MPUC patients without distal metastasis faced a higher risk of CSM (HR = 1.33, 95% CI = 1.10‐1.61, *P* < .0001). However, in the OM analysis, the *P* value of N stage in the test for interaction was .0521 and near .05. This might have resulted from a relatively insufficient sample size or lymph node metastasis might interact with MPUC histology.

## 4 DISCUSSION

In this study, we aimed to investigate prognostic values of clinicopathological characteristics and survival outcomes in MPUC of the urinary bladder. Given that TCC accounts for approximately 95% of bladder cancers, MPUC, and TCC were compared using records from the SEER database according to specified inclusion criteria. Micropapillary urothelial carcinoma and TCC had different effects on patients' OM, especially the 5‐year survival status (*P* < .0001). Moreover, patients with MPUC were at a higher risk of OM (HR = 1.39, 95% CI = 1.22‐1.57, *P* < .0001), but their CSM (HR = 1.18, 95% CI = 1.00‐1.40, *P* = .0505) was fair. This indicated that MPUC could be an independent prognostic factor for OM in patients with urinary bladder cancer. Furthermore, in the subgroup analysis, only MPUC patients without distal metastasis faced a higher risk of CSM (HR = 1.33, 95% CI = 1.10‐1.61, *P* < .0001).

This study supported previous research that reported that MPUC could be an independent prognostic factor for OM (HR = 1.39, 95% CI = 1.22‐1.57, *P* < .0001). However, in terms of CSM, MPUC as a prognostic factor is still controversial because the *P* value for both the adjusted model II (.0505) and the PSM adjusted model (.0469) are near .05 in different sides. Sui et al analyzed 869 MPUC patients from the National Cancer Database (2004‐2014) and suggested that MPUC independently predicted a decreased OM, but they did not analyze CSM.^8^ Vourganti et al enrolled 120 MPBC patients from the SEER database (2001‐2008) and found no survival difference between MPUC and UC after controlling for stage and grade.^11^ A multi‐institutional analysis based on 151 MPUC patients demonstrated that MPUC was not independently associated with the risks of recurrence or OM.^9^ Although Abufaraj et al included 15 studies in their meta‐analysis, their results focused on patients undergoing RC or neoadjuvant chemotherapy.^10^ Compared with the above studies, the present study analyzed a sufficient number of patients and adjusted covariates to consider overall survival. Briefly, this study indicated that MPUC is an independent prognostic factor for OM at the population level. Interestingly, in the subgroup analysis of this study, MPUC patients without distal metastasis faced a higher risk of CSM (HR = 1.33, 95% CI = 1.10‐1.61, *P* < .0001), even though CSM, as an independent prognostic factor, remains controversial. This finding indicated that M0 MPUC patients may require aggressive treatments to improve CSM.

Most MPUC cases in this study were diagnosed with muscle invasive bladder cancer (MIBC) compared with TCC cases (63.39% vs 9.39%, *P* < .001), and MPUC patients had higher rates of lymph node metastasis (24.28% vs 1.32%, *P* < .001) and distal metastasis (10.79% vs 1.17%, *P* < .001). Higher‐grade disease was also more common in the MPUC group (97.49% vs 49.38%, *P* < .001). These aggressive pathologic features have also been noted in other studies.^8^, ^9^, ^12^ According to another study, even if MPUC constituted less than 10% of a UC, the patients had worse prognoses.^13^ Micropapillary urothelial carcinoma contains small, tight clusters of high‐grade or infiltrating tumor cells,^5^ and its morphological characteristics are related directly to molecular alterations.^6^ Conventional TCC may be categorized broadly into the luminal and basal types, but MPUC tends to be luminal with expression of markers such as FOXA1 and GATA3.^14^ Moreover, human epidermal growth factor receptor 2 overexpression and amplification are common in MPUC,^7^, ^15^ which can also be characterized by activation of the miR‐296 and RUVBL1 target genes.^5^ Furthermore, epithelial membrane antigen and E‐cadherin participate in MPUC polarity reversal.^16^ These molecular mechanisms might be helpful for early diagnosis and further treatment of this cancer.

In this study, the most common surgery type in the MPUC group was TURBT (47.01%, 244/519), followed by RC (19.27%, 100/519) and pelvic exenteration (17.34%, 90/519). In addition, MPUC patients had more lymph nodes removed (38.54% vs 4.24%, *P* < .001) and were more likely to be treated with beam radiation (11.18% vs 2.00%, *P* < .001) than patients in the TCC group. Given that 63.39% of MPUC patients have MIBC, the treatment is relatively conservative and may be responsible for worse prognoses. Although RC was recommended in MIBC guidelines, early RC in non‐MIBC patients is still controversial.^10^ A population‐based study showed no difference between RC and bladder preservation surgery for cT1 MPUC,^8^ while Willis et al reported a better prognosis after early RC.^17^ Cisplatin‐based neoadjuvant therapy was commonly given to improve MIBC prognoses, but it is unclear whether it actually does. Sui et al found no survival benefit from MPUC after neoadjuvant chemotherapy.^8^ Although other studies reported pathological downstaging, this does not translate into better survival outcomes.^18^ In this study, MPUC was found to be an independent prognostic factor for OM and for CSM in the M0 subgroup. We suggest that, once MPUC components are found by biopsy, an advanced combined treatment should be considered. However, multicenter clinical trials are needed to establish a better therapeutic protocol for this rare, but aggressive, cancer.

This study had several strengths. First, we enrolled 519 patients with MPUC of the urinary bladder from 2004 to 2016; thus, we had a sufficient sample size to perform an exact and multiform analysis. Subgroup analyses and PSM were used to analyze potential confounding factors. Second, we updated clinicopathological characteristics and survival outcomes of MPUC, based on recent data. However, our study also had some limitations. First, this study had instinct limitations due to its retrospective nature. Selection bias may exist, which is inevitable for clinical observational studies, even those using PSM. Second, the SEER database lacked some essential variables; for example, treatment regimens were classified into two major categories as either surgery or radiotherapy, but chemotherapy and new therapies such as checkpoint‐inhibitor drugs were absent; including these treatment modalities might have led to different outcomes.

## 5 CONCLUSIONS

The prognosis of MPUC is poorer than that of TCC. Micropapillary urothelial carcinoma is an independent prognostic factor for OM in patients with urinary bladder cancer. In the subgroup analysis, only MPUC patients without distal metastasis faced a higher risk of CSM.

## CONFLICT OF INTEREST

Nonfinancial associations that may be relevant to the submitted manuscript.

## AUTHORS' CONTRIBUTION

The first three authors contributed equally as first authors of this manuscript. All authors contributed to the design of the project, data collection and analysis, and contributed to the final manuscript. All authors have read and approved the final submitted manuscript.

## Supporting information

Fig S1

Fig S1Legend

## Data Availability

Publicly available datasets were analyzed in this study. This data can be found here: https://seer.cancer.gov/data/.
