# Supplementary figures and images for: Prognostic values of the clinicopathological characteristics and survival outcomes in micropapillary urothelial carcinoma of the bladder: A SEER database analysis
Source: Cancer Med. 2020 Jun 11;9(14):4897–906. doi: 10.1002/cam4.3147 (PMC7367637; doi:10.1002/cam4.3147)

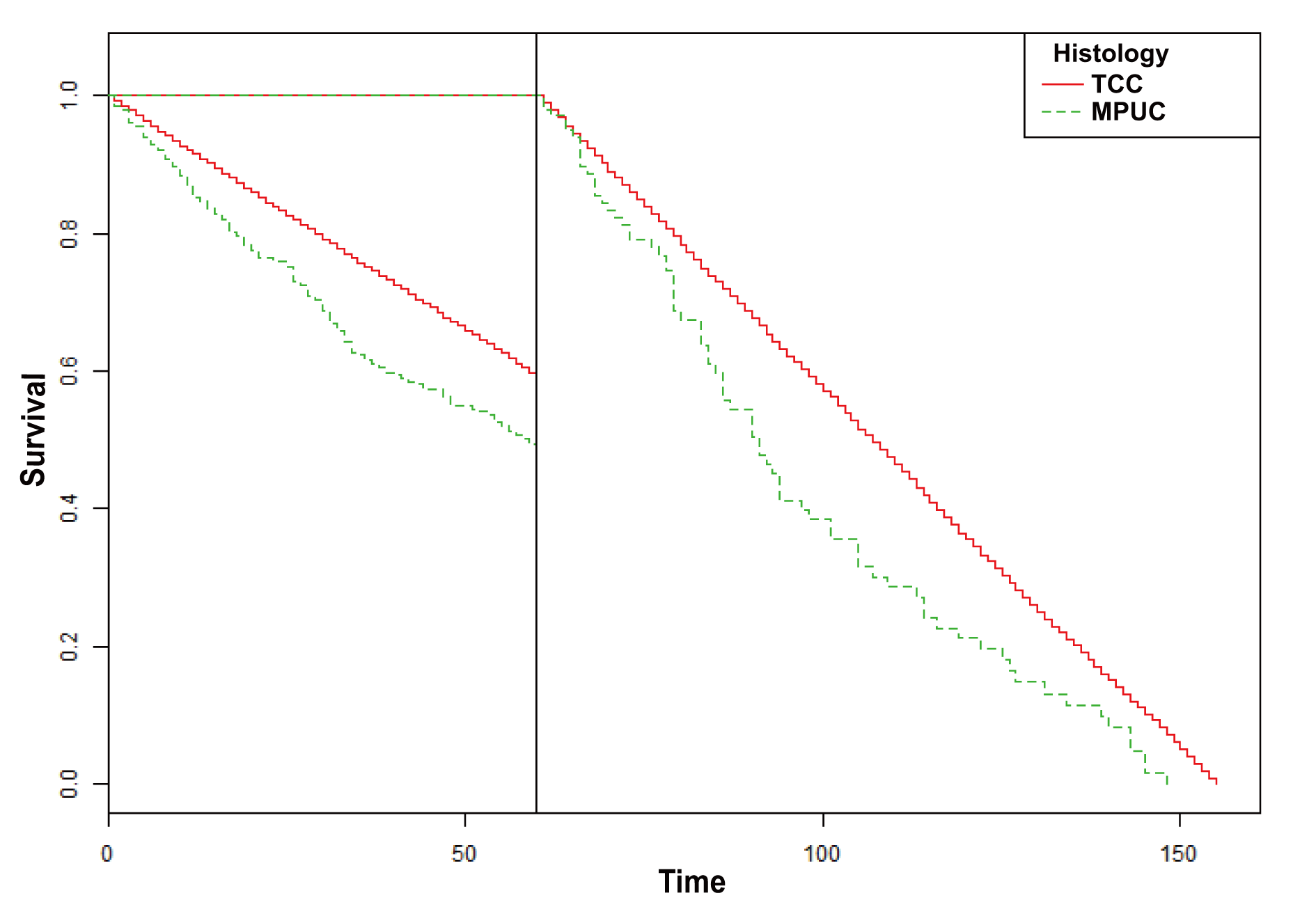

Supplement: Supplementary file 1 — Fig S1 [file CAM4-9-4897-s001.tif]
