# Supplementary material for: Prognostic values of the clinicopathological characteristics and survival outcomes in micropapillary urothelial carcinoma of the bladder: A SEER database analysis
Source: Cancer Med. 2020 Jun 11;9(14):4897–906. doi: 10.1002/cam4.3147 (PMC7367637; doi:10.1002/cam4.3147)
Supplement: Supplementary file 2 — Fig S1Legend [file CAM4-9-4897-s002.docx]

**Figure S1.** 5 years cut of overall mortality of patients with MPUC.
